# Long Chain Omega-3 Polyunsaturated Fatty Acids Improve Vascular Stiffness in Abdominal Aortic Aneurysm: A Randomized Controlled Trial

**DOI:** 10.3390/nu13010138

**Published:** 2020-12-31

**Authors:** Lara T. Meital, Karl Schulze, Rebecca Magee, Jill O’Donnell, Pankaj Jha, Chaim Y. Meital, Rebecca Donkin, Tom G. Bailey, Christopher D. Askew, Fraser D. Russell

**Affiliations:** 1 Centre for Genetics, Ecology & Physiology, University of the Sunshine Coast, Maroochydore, QLD 4556, Australia; Lara.Meital@research.usc.edu.au; 2 School of Health and Behavioural Sciences, University of the Sunshine Coast, Maroochydore, QLD 4556, Australia; rdonkin@usc.edu.au (R.D.); CAskew@usc.edu.au (C.D.A.); 3 Sunshine Vascular, Buderim, QLD 4556, Australia; karl.svi@bigpond.com; 4 Department of Surgery, Sunshine Coast University Hospital, Birtinya, QLD 4575, Australia; r.magee@ausdoctors.net (R.M.); Jill.ODonnell@health.qld.gov.au (J.O.); Pankaj.Jha@health.qld.gov.au (P.J.); 5 Moffat Beach Family Medical Practice, Moffat Beach, QLD 4551, Australia; cmeital@bigpond.com; 6 Physiology and Ultrasound Laboratory in Science and Exercise (PULSE), Centre for Research on Exercise, Physical Activity and Health, School of Human Movement and Nutrition Sciences, The University of Queensland, Queensland, QLD 4343, Australia; Tom.Bailey@uq.edu.au; 7 School of Nursing, Midwifery and Social Work, The University of Queensland, Queensland, QLD 4343, Australia; 8 VasoActive Research Group, Sunshine Coast Health Institute, Sunshine Coast Hospital and Health Service, Birtinya, QLD 4575, Australia

**Keywords:** abdominal aortic aneurysm, pulse wave velocity, vascular stiffness, long chain omega-3 polyunsaturated fatty acids

## Abstract

Abdominal aortic aneurysm (AAA) is a vascular disease involving permanent focal dilation of the abdominal aorta (≥30 mm) that can lead to catastrophic rupture. Destructive remodeling of aortic connective tissue in AAA contributes to wall stiffening, a mechanical parameter of the arterial system linked to a heightened risk of cardiovascular morbidity and mortality. Since aortic stiffening is associated with AAA progression, treatment options that target vascular inflammation would appear prudent. Given this, and growing evidence indicating robust anti-inflammatory and vasoprotective properties for long chain omega-3 polyunsaturated fatty acids (LC n-3 PUFAs), this study evaluated the impact of these nutrients (1.8 g/day for 12 weeks) on indices of vascular stiffness in patients with AAA. At baseline, pulse wave velocity (PWV) and augmentation index normalized to a heart rate of 75 bpm (AIx75) were significantly higher in patients with AAA compared to control participants (PWV: 14.2 ± 0.4 m.s^−1^ vs. 12.6 ± 0.4 m.s^−1^, *p* = 0.014; AIx75: 26.4 ± 1.7% vs. 17.3 ± 2.7%, *p* = 0.005). Twelve-week LC n-3 PUFA supplementation significantly decreased PWV (baseline: 14.2 ± 0.6 m.s^−1^, week 12: 12.8 ± 0.7 m.s^−1^, *p* = 0.014) and heart rate (baseline: 63 ± 3 bpm, week 12: 58 ± 3 bpm, *p* = 0.009) in patients with AAA. No change was observed for patients receiving placebo capsules. While this raises the possibility that LC n-3 PUFAs provide improvements in aortic stiffness in patients with AAA, the clinical implications remain to be fully elucidated.

## 1. Introduction

Abdominal aortic aneurysm (AAA) is a clinically silent cardiovascular disease characterized by permanent and progressive full thickness dilation of the abdominal aortic segment [1]. AAA affects between 1.2 and 3.3% of men over the age of 60 years and, as yet, no proven pharmacological therapies exist to halt or reverse disease progression [2]. AAA is associated with inflammation, elastin degradation and changes to collagen microarchitecture [3,4,5]. Aberrations in vascular extracellular matrix contribute to the development of wall stiffening, a mechanical parameter of the arterial system linked to a heightened risk of systolic hypertension, coronary artery disease, heart failure and stroke [6]. Vascular stiffening, defined as a decrease in vessel distensibility, results in reduced buffering capacity of the vasculature against pulsatile flow from the heart [7]. Alterations in vessel stiffness can be monitored using non-invasive techniques that measure the speed of forward traveling pulse waves (carotid to femoral pulse wave velocity, PWV) [8] or that analyze arterial pressure wave reflection characteristics (pulse wave analysis, PWA) [9]. The latter provides an augmentation index (AIx), a parameter that reflects aortic pressure augmentation relative to pulse pressure [10]. AIx is influenced by the structure and degree of compliance of vessels distal to measurement sites [11].

Factors contributing to vascular stiffening include increasing age, elevated arterial pressure and asymmetry in the occurrence of the major scaffolding proteins collagen and elastin [12]. In patients with AAA, collagen overproduction, diminution of elastin and abnormal calcium deposition in the aortic media moderate vascular stiffening [13,14]. Vascular stiffening in ageing individuals is associated with a significantly higher risk of cardiovascular events and cardiovascular and all-cause mortality [15]. Moreover, in patients with AAA, vascular stiffening has been associated with poor aneurysm shrinkage and negative long-term cardiovascular outcomes following endovascular aneurysm repair [16].

The association between vascular stiffening and inflammation involves (i) impaired vasodilatory responses by inflammatory cytokines [17], (ii) vessel wall calcification [18] and (iii) extracellular matrix degradation [19]. In primary care, inflammation is typically monitored through the assessment of quantifiable blood-associated markers (C-reactive protein, erythrocyte sedimentation rate and plasma viscosity) [20]. In experimental settings, red blood cell distribution width (RDW) is emerging as a novel and reliable biomarker of inflammation and cardiovascular and all-cause mortality. RDW is a measure of size variability or heterogeneity of volume among circulating erythrocytes [21]. Findings from a large cohort study support the existence of strong graded relationships between RDW and both high sensitivity C-reactive protein and erythrocyte sedimentation rate, independent of confounding factors [22]. RDW consistently predicts cardiac mortality [23] and a large meta-analysis has associated each 1% positive increment in RDW with a 14% increase in total mortality risk (HR 1.14; CI 1.11–1.17) [24]. In addition, RDW values above 14% are associated with negative impacts on erythrocyte deformability that can result in impaired RBC flow within the microvasculature [24]. 

Improving vascular stiffness through pharma-nutritional supplementation is a growing area of interest. Our group recently reported a significant decrease in RDW in patients with AAA following 12-week supplementation with 1.8 g long chain omega-3 polyunsaturated fatty acids (LC n-3 PUFAs) [25]. LC n-3 PUFAs, particularly docosahexaenoic acid (C22:6n-3; DHA) and eicosapentaenoic acid (C20:5n-3, EPA), down-regulate multiple aspects of the inflammatory process [26] and reduce the risk of cardiovascular and chronic, age-related disease states [27,28,29]. The anti-inflammatory and vasoprotective properties of LC n-3 PUFAs highlight these nutrients as a potential therapeutic strategy to reverse increased PWV in patients with AAA. The aims of this study were to (i) compare aortic stiffness in patients with AAA and control participants, (ii) assess the relationship between RDW and PWV in patients with AAA, and (iii) evaluate the impact of LC n-3 PUFA supplementation on vascular stiffness parameters in patients with AAA.

## 2. Materials and Methods 

Vascular stiffness was evaluated in men with AAA (*n* = 30), recruited for an observational (case-control) study [25] and compared to a subset of healthy male participants (*n* = 20) involved in the same study (Table 1). The impact of LC n-3 PUFA supplementation on vascular stiffness in the AAA population was investigated as a sub-study of a previously described double-blind, placebo-controlled trial (ANZCTR12616000483459) [25]. Patients received LC n-3 PUFA capsules (1.8 g; 5:1 ratio of DHA:EPA; *n* = 15) or placebo capsules (49:49:2 ratio of corn oil, olive oil and fish oil; *n* = 15), for 12 weeks (for trial details, including evaluation of patient compliance, see [25]). Patients were included if they were male with a small AAA < 5 cm. Exclusion criteria were consumption of ≥3 fish meals per week, the use of fish oil or krill oil supplements, age < 60 years or >86 years, BMI > 39 kg·m^−2^, cardiac arrhythmia, heart failure, symptomatic aortic stenosis, or chronic obstructive pulmonary disease. A family history of AAA or known aneurysmal disease served as additional exclusion criteria for control participants. Vascular stiffness, heart rate and central blood pressure indices were assessed by two operators at baseline, week 3 and week 12 using the SphygmoCor Xcel (SphygmoCor ver 6.31, AtCor Medical Pty. Ltd., Sydney, Australia) and a previously described protocol [30]. RDW was measured in whole blood as part of full blood count analyses. Measurements were obtained within 10 min of blood collections using a Coulter A^C^·T diff™ Analyzer, as previously described [25]. The omega-3 index, a measurement indicative of long-term fatty acid intake, was assessed in red blood cells of healthy controls at baseline and in red blood cells of patients with AAA at baseline and at weeks 3 and 12, as previously described [25]. Continuous demographic data for patients with AAA and control participants were compared using a Student’S *t*-test and are presented as mean ± SD. Categorical demographic variables were compared using a Fisher’s exact test. Experimental data are presented as mean ± SEM and between group differences were examined by student’s *t*-test analysis. The association between PWV and AAA was assessed using linear regression analysis with adjustment for covariates shown to be imbalanced between groups (low-dose aspirin, smoking history). Group size estimates for the observational study were based on PWV values reported in healthy controls (PWV, 10.0 ± 1.7, *n* = 20) and patients with AAA (PWV, 14.8 ± 4.9, *n* = 18) [31]. A group size estimate of 9 was calculated with 80% power (alpha level of 0.05) using power/sample size (Univ. British Columbia) and pooled variance (Solvers statistics) calculators. Data were analyzed with Prism (GraphPad Software, La Jolla, CA, USA). IBM SPSS Statistics Version 24 was used for multi-variable regression analysis and statistical significance was set at *p* < 0.05.

## 3. Results

### 3.1. Vascular Stiffness Indices Are Elevated in Patients with Abdominal Aortic Aneurysm 

PWV and AIx75 measurements were significantly higher in patients with AAA compared to the healthy control cohort (PWV: 14.2 ± 0.4 m.s^−1^ vs. 12.6 ± 0.4 m.s^−1^, *p* = 0.014; AIx75: 26.4 ± 1.7% vs. 17.3 ± 2.7%, *p* = 0.005).

### 3.2. AAA Is a Significant Independent Determinant of PWV

Linear regression analysis highlighted a significant association between PWV and AAA (β = 0.463, 95% CI: 0.426 to 3.248, *p* = 0.012) following adjustment for covariates shown to be imbalanced between the control and AAA cohorts (smoking: β = −0.024, 95% CI: −1.618 to 1.394, *p* = 0.881 and low-dose aspirin: β = −0.152, 95% CI: −1.946 to 0.754, *p* = 0.376).

### 3.3. PWV Correlates with Omega-3 Index and Red Blood Cell Distribution Width (RDW) in Patients with AAA 

**Figure 1:**
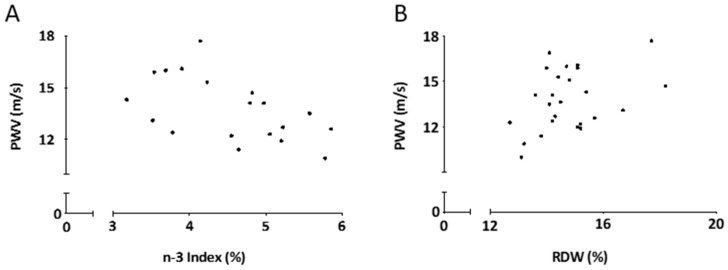
Relationship between pulse wave velocity (PWV) and omega-3 index (**A**) and PWV and red blood cell distribution width (RDW) (**B**) at baseline in patients with AAA.

A significant negative correlation was observed between PWV and omega-3 index (Figure 1A; *r* = −0.54, *p* = 0.017) and a significant positive correlation was observed between PWV and RDW (Figure 1B; *r* = 0.40, *p* = 0.047) in patients with AAA at baseline. 

### 3.4. Long-Chain Omega-3 PUFA Supplementation Improves Vascular Stiffness in Patients with AAA

Twelve-week LC n-3 PUFA supplementation significantly decreased PWV (*p* = 0.014) and heart rate (*p* = 0.009) in patients with AAA (Table 2). Remaining vascular stiffness and central blood pressure indices were unaffected by LC n-3 PUFA supplementation. No changes in vascular stiffness parameters were observed for the placebo group.

## 4. Discussion

Vascular stiffness is increased in patients with AAA compared to healthy adults of a similar age. This finding is concordant with data arising from studies involving similarly aged aneurysm patients [30,31]. AAA confers a cardiovascular comorbidity burden prior to surgical intervention and a higher risk of adverse cardiovascular events in the 5 years following elective surgery [32]. In view of this, treatments that improve vascular stiffening are likely to positively impact long-term survival in this patient population. 

In this study, twelve-week supplementation with LC n-3 PUFAs (1.5 g DHA, 0.3 g EPA delivering a total of 1.8 g LC n-3 PUFAs per day) significantly decreased PWV in patients with AAA. This finding is consistent with a large community-based study involving similarly aged individuals without AAA [33]. In that study, carotid-femoral PWV was assessed in a cohort of adults with multiple co-morbidities (*n* = 3055, mean age 66 ± 9 years). Participants did not receive LC n-3 PUFA supplementation, however, differences in background fatty acid levels were noted through assessment of the omega-3 index. The results indicated that higher omega-3 index values were associated with lower carotid-femoral PWV following age-and sex-adjusted analyses (*r* = −0.098, *p* < 0.001) and following multivariable adjusted analyses (*r* = −0.060, *p* = 0.002). This is concordant with the current study where a significant negative correlation was observed between PWV and omega-3 index in patients with AAA. 

Although the precise mechanisms underlying LC n-3 PUFA-mediated improvements in vascular stiffness remain to be established, their beneficial effects on cardiovascular health are reported to be a consequence of abundant accumulation in the sn-2 position of membrane phospholipids. In this location, LC n-3 PUFAs compete with arachidonic acid (an omega-6 fatty acid) for enzymes involved in the biosynthesis of pro-inflammatory mediator molecules [34]. The subsequent metabolism of LC n-3 PUFAs is known to produce an alternate series of less biologically potent eicosanoids with weaker pro-inflammatory, platelet-aggregating and vasoconstrictive activities [35,36]. Our finding of a significant positive correlation between PWV and RDW is concordant with this mechanism. In view of this, it is possible that LC n-3 PUFA-mediated decreases in vascular stiffness reflect an improved inflammatory status among patients with AAA who received this supplement. An additional mechanism has been reported. A recent meta-analysis indicated a robust protective effect of LC n-3 PUFAs on endothelial function [15]. In that study, supplementation with 0.45 to 4.5 g/day of LC n-3 PUFAs was associated with a 2.3% increase in flow mediated dilation (FMD). Smaller FMD is an indicator of endothelial dysfunction, a factor that contributes to vascular stiffness and predicts a heightened risk of adverse cardiovascular events [37].

Age, sex, systolic blood pressure and heart rate are determinants of PWV. An effect of heart rate on PWV has been described previously in Sprague-Dawley rats that were fitted with a right atrial pacing electrode [38]. The study showed that animals paced at low heart rates had significantly lower PWV than animals that were paced at higher heart rates. The positive correlation persisted after correction for mean arterial blood pressure. In our study, heart rate was significantly reduced after 12-week supplementation with LC n-3 PUFA capsules, raising the possibility of an indirect effect of LC n-3 PUFAs on PWV involving modulation of heart rate.

Older populations have a higher PWV than younger populations [39,40,41] and men over 30 years have a higher PWV than women who are over 30 years when matched for age [39]. Only male participants were recruited to this study, and patients with AAA were matched for age with control participants. That only men were recruited to the study is both a strength and a limitation. Male sex was an inclusion criterion, thus negating the potential confounding influence of sex on PWV determinations. Nonetheless, when examining the effect of LC n-3 PUFA supplementation on PWV, our findings are applicable to men only and further studies will be required to determine whether the results are translatable to women with AAA. A positive correlation exists for systolic blood pressure(SBP) and PWV [39], with a 0.073 m.s^−1^ increase in PWV reported for each mmHg increase in SBP [40]. In our study, SBP (and diastolic blood pressure(DBP)) were similar across groups in both the observational study and the omega-3 clinical trial.

Discrepancies exist in the literature regarding the effect of LC n-3 PUFAs on PWV. Where an effect of LC n-3 PUFAs has been reported, participant age was found to be a determinant of efficacy. In a study recruiting healthy participants, daily intake of 1.86 g EPA and 1.5 g DHA for 12 weeks caused a significant (−9%) reduction in carotid-femoral PWV in older participants (60–80 years), but provided no benefit in a younger cohort (21–35 years) [41]. A trend for lower PWV was detected in patients with hypertension receiving 3.36 g LC n-3 PUFAs per day for 12 weeks compared to a placebo; however, this result did not reach significance. It is noteworthy that the age of patients recruited to that study (mean, 61.1 years) was lower than the age of patients in our study (Table 1), and it is possible that younger age groups are less amenable to the effects of LC n-3 PUFA supplementation. Limitations of this study included the absence of ultrasound screening for AAA in control participants, small sample size, and a single intervention dose. Several recent trials of omega-3 fatty acids reported no clinical benefit of omega-3 fatty acid supplementation in patients who have cardiovascular disease. In a large observational study of older Danish men with AAA, no correlation was detected between omega-3 index and maximal aortic diameter or AAA growth rate [42]. High background intake of omega-3 fatty acids is a likely contributor to neutral findings [43]. Danish populations have a higher intake of seafood-derived omega-3 fatty acids than Australian populations (1225 mg/day compared to 286 mg/day) [44]. In addition, the consumption of ≥3 fish meals per week was an exclusion criterion for enrolment in our study. In the Danish study, the baseline omega-3 index was considerably higher than that identified in the current study of enrolled Australian participants (7.6% compared to 4.37–4.53%).

## 5. Conclusions

This study observed elevated PWV values in patients with AAA compared to a healthy control cohort. A twelve-week LC n-3 PUFA supplementation regimen lowered AAA patient aortic stiffness and resting heart rate to levels that were comparable to the control cohort. While these improvements are likely to be beneficial in terms of risks associated with cardiovascular morbidity and mortality, the clinical implications remain to be fully elucidated.

## Figures and Tables

**Table 1:** Demographic, biometric and medical characteristics of male patients with abdominal aortic aneurysm (AAA) and control participants. Continuous demographic data are presented as mean ± SD, categorical demographic data are presented as number (percentage).

	Observational Study		Omega-3 Clinical Trial	
Variable	Control Participants (*n* = 20)	AAA Patients (*n* = 30)	AAAn-3 Cohort (*n* = 15)	AAA Placebo Cohort (*n* = 15)
Age (years)	73.2 ± 5.6	74.4 ± 5.3	73.6 ± 5.0	75.1 ± 5.7
AAA size (mm)		39.3 ± 5.3	39.3 ± 5.7	39.2 ± 5.0
Smoking:				
Never	8 (40%)	3 (10%) †	1 (7%)	2 (13%)
Past	11 (55%)	24 (80%)	12 (80%)	12 (80%)
Current	1 (5%)	3 (10%)	2 (13%)	1 (7%)
BMI (kg/m^2^)	28.4 ± 5.3	29.5 ± 5.2	29.4 ± 4.1	29.0 ± 5.0
SBP (mmHg)	141 ± 17	140 ± 18	136 ± 16	144 ± 20
DBP (mmHg)	81 ± 7	79 ± 10	78 ± 8	80 ± 11
Hypertension	12 (60%)	17 (57%)	6 (40%)	11 (73%)
Diabetes	1 (5%)	4 (13%)	3 (20%)	1 (7%)
Dyslipidemia	15 (75%)	19 (63%)	7 (47%)	12 (80%)
CHD	6 (30%)	9 (30%)	5 (33%)	4 (27%)
Anti-hypertensives				
Beta blockers	3 (15%)	7 (23%)	4 (27%)	3 (20%)
ARBs	3 (15%)	8 (27%)	3 (20%)	5 (33%)
ACE inhibitors	7 (35%)	4 (13%)	3 (20%)	1 (7%)
CCBs	2 (10%)	6 (20%)	1 (7%)	5 (33%)
Diuretics	2 (10%)	3 (10%)	2 (13%)	1 (7%)
Anti-platelet drugs	3 (15%)	18 (60%) †	11 (73%)	7 (47%)
NSAIDs	1 (5%)	3 (10%)	1 (7%)	2 (13%)
Statins	14 (70%)	21 (70%)	8 (53%) ‡	13 (87%)
Baseline omega-3 Index			4.53 ± 0.22	4.37 ± 0.20
12-Week omega-3 Index			8.03 ± 0.20	4.26 ± 0.28

† AAA significantly different to control (Fisher’s exact test, *p* < 0.05). ‡ AAA omega-3 cohort significantly different to placebo cohort (Fisher’s exact test, *p* < 0.05). ARBs, Angiotensin receptor blockers; BMI, body mass index; CHD, coronary heart disease; CCBs, Calcium channel blockers; NSAIDs, non-steroidal anti-inflammatory drugs; SBP, systolic blood pressure; DBP, diastolic blood pressure.

**Table 2:** Vascular stiffness, heart rate and central blood pressure indices at baseline and weeks 3 and 12 post treatment (mean ± SEM).

Variable	Fish Oil Cohort			Placebo Cohort		
	Baseline	Week 3	Week 12	Baseline	Week 3	Week 12
PWV (ms^−1^)	14.2 ± 0.6	13.8 ± 1.2	12.8 ± 0.7 *	14.6 ± 0.6	14.7 ± 0.6	14.0 ± 0.5
AIx75 (%)	26.4 ± 3.0	24.0 ± 4.2	23.7 ± 2.5	28.3 ± 2.2	28.6 ± 2.6	28.9 ± 2.5
RM (%)	64.1 ± 2.2	61.5 ± 3.0	66.0 ± 3.3	64.7 ± 2.3	67.9 ± 3.3	67.8 ± 1.6
HR (bpm)	63 ± 3	59 ± 3	58 ± 3 **	66 ± 2	65 ± 2	66 ± 3
cSBP (mmHg)	126 ± 4	127 ± 5	124 ± 3	129 ± 4	129 ± 4	126 ± 3
cDBP (mmHg)	80 ± 3	79 ± 4	79 ± 3	81 ± 3	83 ± 3	79 ± 3
cPP (mmHg)	46 ± 3	52 ± 5	45 ± 2	49 ± 3	46 ± 3	47 ± 2

Fish oil cohort week 12 significantly different to fish oil cohort baseline (* *p* < 0.05, ** *p* < 0.01). PWV, pulse wave velocity; AIx75, heart rate corrected augmentation index; RM, reflection magnitude; HR, aortic heart rate; cSBP, central systolic blood pressure; cDBP, central diastolic blood pressure; cPP, central pulse pressure.

## Data Availability

All data are available at Figshare knowledge at Figshare.com and can be accessed at: 10.6084/m9.figshare.13191464.

## References

[1] Raffort J., Lareyre F., Clément M., Hassen-Khodja R., Chinetti G., Mallat Z. (2017). Monocytes and macrophages in abdominal aortic aneurysm. Nat. Rev. Cardiol..

[2] Owens D.K., Davidson K.W., Krist A.H., Barry M.J., Cabana M., Caughey A.B., Doubeni C.A., Epling J.W., Kubik M., Landefeld C.S. (2019). Screening for abdominal aortic aneurysm: US Preventive Services Task Force recommendation statement. JAMA.

[3] Wales K.M., Kavazos K., Nataatmadja M., Brooks P.R., Williams C., Russell F.D. (2014). N-3 PUFAs protect against aortic inflammation and oxidative stress in angiotensin II-infused apolipoprotein E^-/-^ mice. PLoS ONE.

[4] Kavazos K., Nataatmadja M., Wales K.M., Hartland E., Williams C., Russell F.D. (2015). Dietary supplementation with omega-3 polyunsaturated fatty acids modulate matrix metalloproteinase immunoreactivity in a mouse model of pre-abdominal aortic aneurysm. Heart Lung Circ..

[5] Meital L.T., Windsor M.T., Maynard A.E., Schulze K., Magee R., O’Donnell J., Jha P., Meital C.Y., Perissiou M., Coverdale S. (2020). Endotoxin tolerance in abdominal aortic aneurysm macrophages, in vitro: A case–control study. Antioxidants.

[6] Shirwany N.A., Zou M.-h. (2010). Arterial stiffness: A brief review. Acta Pharmacol. Sin..

[7] Lacolley P., Regnault V., Segers P., Laurent S. (2017). Vascular smooth muscle cells and arterial stiffening: Relevance in development, aging, and disease. Physiol. Rev..

[8] McManus S., Tejera N., Awwad K., Vauzour D., Rigby N., Fleming I., Cassidy A., Minihane A.M. (2016). Differential effects of EPA versus DHA on postprandial vascular function and the plasma oxylipin profile in men. J. Lipid Res..

[9] Perrault R., Omelchenko A., Taylor C.G., Zahradka P. (2019). Establishing the interchangeability of arterial stiffness but not endothelial function parameters in healthy individuals. BMC Cardiovasc. Disord..

[10] Pase M.P., Grima N.A., Sarris J. (2011). The effects of dietary and nutrient interventions on arterial stiffness: A systematic review. Am. J. Clin. Nutr..

[11] Butlin M., Qasem A. (2016). Large artery stiffness assessment using SphygmoCor technology. Pulse.

[12] Boutouyrie P., Bruno R.-M. (2019). The clinical significance and application of vascular stiffness measurements. Am. J. Hypertens..

[13] Dobrin P.B. (1989). Pathophysiology and pathogenesis of aortic aneurysms: Current concepts. Surg. Clin. N. Am..

[14] Russo L. (2006). Thoracic aortic compliance as a determinant of rupture of abdominal aortic aneurysms. Ann. N. Y. Acad. Sci..

[15] Vlachopoulos C., Aznaouridis K., Stefanadis C. (2010). Prediction of cardiovascular events and all-cause mortality with arterial stiffness: A systematic review and meta-analysis. J. Am. Coll. Cardiol..

[16] Kanamoto R., Otsuka H., Anegawa T., Takaseya T., Shintani Y., Tobinaga S., Onitsuka S., Ueno M., Hiromatsu S., Tanaka H. (2019). P5601 High pulse wave velocity is associated with poor shrinkage of abdominal aortic aneurysm in endovascular aneurysm repair patients. Eur. Heart J..

[17] Wilkinson I.B., MacCallum H., Cockcroft J.R., Webb D.J. (2002). Inhibition of basal nitric oxide synthesis increases aortic augmentation index and pulse wave velocity in vivo. Br. J. Clin. Pharmacol..

[18] Buendía P., de Oca A.M., Madueño J.A., Merino A., Martín-Malo A., Aljama P., Ramírez R., Rodríguez M., Carracedo J. (2015). Endothelial microparticles mediate inflammation-induced vascular calcification. FASEB J..

[19] Jain S., Khera R., Corrales–Medina V.F., Townsend R.R., Chirinos J.A. (2014). Inflammation and arterial stiffness in humans. Atherosclerosis.

[20] Watson J., Jones H.E., Banks J., Whiting P., Salisbury C., Hamilton W. (2019). Use of multiple inflammatory marker tests in primary care: Using Clinical Practice Research Datalink to evaluate accuracy. Br. J. Gen. Pract..

[21] Salvagno G.L., Sanchis-Gomar F., Picanza A., Lippi G. (2015). Red blood cell distribution width: A simple parameter with multiple clinical applications. Crit. Rev. Clin. Lab. Sci..

[22] Lippi G., Targher G., Montagnana M., Salvagno G.L., Zoppini G., Guidi G.C. (2009). Relation between red blood cell distribution width and inflammatory biomarkers in a large cohort of unselected outpatients. Arch. Pathol. Lab. Med..

[23] Montagnana M., Cervellin G., Meschi T., Lippi G. (2012). The role of red blood cell distribution width in cardiovascular and thrombotic disorders. Clin. Chem. Lab. Med..

[24] Patel K.V., Mohanty J.G., Kanapuru B., Hesdorffer C., Ershler W.B., Rifkind J.M. (2013). Association of the Red Cell Distribution Width with Red Blood Cell Deformability. Adv. Exp. Med. Biol..

[25] Meital L.T., Windsor M.T., Jewell R.M.R., Young P., Schulze K., Magee R., O’Donnell J., Jha P., Perissiou M., Golledge J. (2019). n-3 PUFAs improve erythrocyte fatty acid profile in patients with small AAA: A randomized controlled trial. J. Lipid Res..

[26] Meital L.T., Windsor M.T., Perissiou M., Schulze K., Magee R., Kuballa A., Golledge J., Bailey T.G., Askew C.D., Russell F.D. (2019). Omega-3 fatty acids decrease oxidative stress and inflammation in macrophages from patients with small abdominal aortic aneurysm. Sci. Rep..

[27] Del Gobbo L.C., Imamura F., Aslibekyan S., Marklund M., Virtanen J.K., Wennberg M., Yakoob M.Y., Chiuve S.E., Dela Cruz L., Frazier-Wood A.C. (2016). ω-3 polyunsaturated fatty acid biomarkers and coronary heart disease: Pooling project of 19 cohort studies. JAMA Intern. Med..

[28] Zheng J., Huang T., Yu Y., Hu X., Yang B., Li D. (2012). Fish consumption and CHD mortality: An updated meta-analysis of seventeen cohort studies. Public Health Nutr..

[29] Wu S., Ding Y., Wu F., Li R., Hou J., Mao P. (2015). Omega-3 fatty acids intake and risks of dementia and Alzheimer’s disease: A meta-analysis. Neurosci. Biobehav. Rev..

[30] Perissiou M., Bailey T.G., Windsor M., Greaves K., Nam M.C., Russell F.D., O’Donnell J., Magee R., Jha P., Schulze K. (2019). Aortic and systemic arterial stiffness responses to acute exercise in patients with small abdominal aortic aneurysms. Eur. J. Vasc. Endovasc. Surg..

[31] Durmus I., Kazaz Z., Altun G., Cansu A. (2014). Augmentation index and aortic pulse wave velocity in patients with abdominal aortic aneurysms. Int. J. Clin. Exp. Med..

[32] Karthikesalingam A., Bahia S., Patterson B., Peach G., Vidal-Diez A., Ray K., Sharma R., Hinchliffe R., Holt P., Thompson M. (2013). The shortfall in long-term survival of patients with repaired thoracic or abdominal aortic aneurysms: Retrospective case–control analysis of Hospital Episode Statistics. Eur. J. Vasc. Endovasc. Surg..

[33] Kaess B.M., Harris W.S., Lacey S., Larson M.G., Hamburg N.M., Vita J.A., Robins S.J., Benjamin E.J., Mitchell G.F., Vasan R.S. (2015). The relation of red blood cell fatty acids with vascular stiffness, cardiac structure and left ventricular function: The Framingham Heart Study. Vasc. Med..

[34] Massaro M., Scoditti E., Carluccio M.A., De Caterina R. (2008). Basic mechanisms behind the effects of n-3 fatty acids on cardiovascular disease. Prostaglandins Leukot. Essent. Fat. Acids.

[35] Yates C.M., Calder P.C., Rainger G.E. (2014). Pharmacology and therapeutics of omega-3 polyunsaturated fatty acids in chronic inflammatory disease. Pharmacol. Therapeut..

[36] Calder P.C. (2015). Marine omega-3 fatty acids and inflammatory processes: Effects, mechanisms and clinical relevance. Biochim. Biophys. Acta Mol. Cell Biol. Lipids.

[37] Brevetti G., Silvestro A., Schiano V., Chiariello M. (2003). Endothelial dysfunction and cardiovascular risk prediction in peripheral arterial disease: Additive value of flow-mediated dilation to ankle-brachial pressure index. Circulation.

[38] Tan I., Butlin M., Liu Y.Y., Ng K., Avolio A.P. (2012). Heart rate dependence of aortic pulse wave velocity at different arterial pressures in rats. Hypertension.

[39] Magalhães P., Capingana D.P., Silva A.B., Ferreira A.V., de Sá Cunha R., Rodrigues S.L., Mill J.G. (2013). Age-and gender-specific reference values of pulse wave velocity for African adults: Preliminary results. Age.

[40] Krantz M.J., Havranek E.P., Pereira R.I., Beaty B., Mehler P.S., Long C.S. (2015). Effects of omega-3 fatty acids on arterial stiffness in patients with hypertension: A randomized pilot study. J. Negat. Results Biomed..

[41] Monahan K.D., Feehan R.P., Blaha C., McLaughlin D.J. (2015). Effect of omega-3 polyunsaturated fatty acid supplementation on central arterial stiffness and arterial wave reflections in young and older healthy adults. Physiol. Rep..

[42] Lindholt J.S., Kristensen K.L., Burillo E., Martinez-Lopez D., Calvo C., Ros E., Martín-Ventura J.L., Sala-Vila A. (2018). Arachidonic acid, but not omega-3 index, relates to the prevalence and progression of abdominal aortic aneurysm in a population-based study of Danish men. J. Am. Heart Assoc..

[43] Bowen K.J., Harris W.S., Kris-Etherton P.M. (2016). Omega-3 fatty acids and cardiovascular disease: Are there benefits?. Curr. Treat. Options Cardio. Med..

[44] Micha R., Khatibzadeh S., Shi P., Fahimi S., Lim S., Andrews K.G., Engell R.E., Powles J., Ezzati M., Mozaffarian D. (2015). Global, regional, and national consumption levels of dietary fats and oils in 1990 and 2010: A systematic analysis including 266 country-specific nutrition surveys. BMJ.

